# Supplementary material for: Impact of the COVID-19 Pandemic on Inpatient Antibiotic Consumption in Switzerland
Source: Antibiotics (Basel). 2022 Jun 11;11(6):792. doi: 10.3390/antibiotics11060792 (PMC9219927; doi:10.3390/antibiotics11060792)
Supplement: Supplementary file 1 [file antibiotics-11-00792-s001.zip › antibiotics-1764854-supplementary.pdf]

Supplementary Materials

Table S1: *Hospital occupancy over time in thousands of bed-days for the 56 hospitals participating in the ANRESIS surveillance database for the entire hospital and the intensive care unit. ICU, intensive care unit;*

	**Entire hospital**			**ICU**		
	**2018**	**2019**	**2020**	**2018**	**2019**	**2020**
**French**	1'094	1'095	1'023	38	38	41
**German**	2'781	2'632	2'438	97	95	93
**Italian**	303	307	269	11	11	14
**Switzerland**	4'178	4'034	3'730	147	144	148

Table S2: *Detailed values for the ITS analysis of the different time periods; data from Figures 3, 4 and S2 .*

**Figure**	**term**	**estimate**	**std.error**	**statistic**	**p.value**
**Figure 3A left**	(Intercept)	-38.148	96.800	-0.394	0.697
	1st wave	5.690	2.870	1.983	0.059
	2nd wave	-2.521	3.269	-0.771	0.448
	Int. phase	-5.004	2.822	-1.773	0.089
	date	0.005	0.005	0.911	0.372
	harmonic(month, 1, 12)1	-0.926	0.932	-0.993	0.331
	harmonic(month, 1, 12)2	-0.963	0.964	-0.999	0.328
**Figure 3A right**	(Intercept)	-52.277	218.135	-0.240	0.813
	1st wave	15.728	6.467	2.432	0.023
	2nd wave	2.251	7.366	0.306	0.763
	Int. phase	-13.728	6.359	-2.159	0.042
	date	0.008	0.012	0.646	0.525
	harmonic(month, 1, 12)1	-2.236	2.101	-1.064	0.298
	harmonic(month, 1, 12)2	-1.234	2.172	-0.568	0.575

Figure	term	estimate	std.error	statistic	p.value
	(Intercept)	-24.801	20.455	-1.212	0.238
	1st wave	1.404	0.606	2.315	0.030
	2nd wave	0.140	0.691	0.203	0.841
**Figure 3B left**	Int. phase	-0.670	0.596	-1.124	0.273
	date	0.002	0.001	1.520	0.142
	harmonic(month, 1, 12)1	-0.404	0.197	-2.052	0.052
	harmonic(month, 1, 12)2	0.055	0.204	0.268	0.791
	(Intercept)	-82.640	100.613	-0.821	0.420
	1st wave	7.130	2.983	2.390	0.025
	2nd wave	1.426	3.397	0.420	0.679
**Figure 3B right**	Int. phase	-3.204	2.933	-1.092	0.286
	date	0.006	0.006	1.053	0.303
	harmonic(month, 1, 12)1	-0.319	0.969	-0.329	0.745
	harmonic(month, 1, 12)2	2.006	1.002	2.002	0.057
	(Intercept)	2.451	3.673	0.667	0.509
	1st wave	0.542	0.212	2.559	0.015
	2nd wave	-0.307	0.182	-1.689	0.100
**Figure 4A**	Int. phase	-0.296	0.175	-1.698	0.098
	date	-0.000	0.000	-0.240	0.812
	harmonic(month, 1, 12)1	0.028	0.059	0.473	0.639
	harmonic(month, 1, 12)2	0.089	0.061	1.473	0.150
	(Intercept)	0.181	0.376	0.480	0.634
	1st wave	0.050	0.022	2.311	0.027
	2nd wave	0.020	0.019	1.076	0.289
**Figure 4B**	Int. phase	-0.009	0.018	-0.496	0.623
	date	-0.000	0.000	-0.170	0.866
	harmonic(month, 1, 12)1	-0.004	0.006	-0.586	0.561
	harmonic(month, 1, 12)2	0.015	0.006	2.429	0.020
	(Intercept)	3.617	4.478	0.808	0.425
**Figure S2 French J01**	1st wave	0.984	0.258	3.806	0.001
	2nd wave	-0.133	0.222	-0.600	0.552

Figure	term	estimate	std.error	statistic	p.value
	Int. phase	-0.217	0.213	-1.022	0.314
	date	-0.000	0.000	-0.503	0.618
	harmonic(month, 1, 12)1	0.027	0.072	0.369	0.714
	harmonic(month, 1, 12)2	0.100	0.074	1.352	0.185
**Figure S2 French BS**	(Intercept)	0.096	0.492	0.195	0.847
	1st wave	0.066	0.028	2.318	0.026
	2nd wave	0.025	0.024	1.048	0.302
	Int. phase	-0.005	0.023	-0.227	0.822
	date	0.000	0.000	0.040	0.968
	harmonic(month, 1, 12)1	-0.003	0.008	-0.422	0.676
	harmonic(month, 1, 12)2	0.013	0.008	1.574	0.125
**Figure S2 German J01**	(Intercept)	2.293	3.143	0.729	0.471
	1st wave	0.330	0.181	1.822	0.077
	2nd wave	-0.341	0.155	-2.193	0.035
	Int. phase	-0.291	0.149	-1.947	0.060
	date	-0.000	0.000	-0.212	0.833
	harmonic(month, 1, 12)1	0.022	0.051	0.438	0.664
	harmonic(month, 1, 12)2	0.073	0.052	1.406	0.169
**Figure S2 German BS**	(Intercept)	0.191	0.345	0.554	0.583
	1st wave	0.025	0.020	1.272	0.212
	2nd wave	0.018	0.017	1.073	0.291
	Int. phase	-0.007	0.016	-0.422	0.675
	date	-0.000	0.000	-0.232	0.818
	harmonic(month, 1, 12)1	-0.004	0.006	-0.770	0.446
	harmonic(month, 1, 12)2	0.014	0.006	2.421	0.021
**Figure S2 Italian J01**	(Intercept)	-2.435	10.690	-0.228	0.821
	1st wave	1.385	0.617	2.246	0.031
	2nd wave	-0.831	0.529	-1.571	0.125
	Int. phase	-0.901	0.508	-1.774	0.085
	date	0.000	0.001	0.410	0.684
	harmonic(month, 1, 12)1	0.138	0.172	0.800	0.429

Figure	term	estimate	std.error	statistic	p.value
	harmonic(month, 1, 12)2	0.303	0.176	1.721	0.094
**Figure S2 Italian BS**	(Intercept)	0.490	1.787	0.274	0.785
	1st wave	0.380	0.103	3.687	0.001
	2nd wave	0.014	0.088	0.154	0.879
	Int. phase	-0.066	0.085	-0.775	0.443
	date	-0.000	0.000	-0.154	0.879
	harmonic(month, 1, 12)1	0.008	0.029	0.263	0.794
	harmonic(month, 1, 12)2	0.052	0.029	1.770	0.085

***Table S3**: Detailed results for the antibiotic consumption analyses of the monthly ANRESIS data. The table shows consumption for each pandemic phase compared to the same periods before the pandemic. PC, pre-COVID-19 period; C, COVID-19 period; Int. period, intermediate period; AB, antibiotics; J01, antibiotics for systemic (ATC category J01); ICU, intensive care unit.*

Hospital	antibiotic (group)	Total			1st wave			2nd wave			Int. period		
		PC	C	%	PC	C	%	PC	C	%	PC	C	%
**Entire hospital**	J01	49.9	49.6	-0.6	49.2	56.5	+14.9	49.7	49.6	-0.2	50.9	48.0	-5.6
	Piperacillin-tazobactam	3.24	3.56	+10.0	3.21	3.91	+21.9	3.21	3.81	+19.0	3.34	3.21	-4.0
	Broad-spectrum	6.23	6.96	+11.7	6.08	7.71	+26.8	6.18	7.27	+17.6	6.35	6.42	+1.2
	Pneumonia AB[1]	26.9	26.1	-3.1	26.0	30.6	+17.5	26.6	26.0	-2.5	27.7	25.2	-9.1
	Carbapenems	1.47	1.81	+22.8	1.36	2.05	+50.9	1.39	1.88	+36.0	1.50	1.64	+8.8
	Fluoroquinolones	0.48	0.45	-7.5	0.36	0.74	+106.1	0.45	0.41	-7.8	0.54	0.37	-30.0
	Macrolides	2.33	1.82	-22.1	2.16	3.24	+49.8	2.39	1.77	-25.8	2.23	1.44	-35.4
	Azithromycin	0.29	0.32	+9.9	0.17	0.45	+158.5	0.29	0.34	+16.7	0.29	0.25	-13.1
	Co-Amoxicillin	15.4	14.3	-7.4	15.2	16.2	+6.4	15.3	13.9	-9.6	15.9	14.4	-9.6
	3rd and 4th generation Cephalosporins	6.46	6.91	+7.1	6.58	7.52	+14.2	6.46	7.12	+10.3	6.59	6.67	+1.2
**ICU**	J01	88.4	90.0	+1.8	84.5	104.5	+23.8	86.6	94.1	+8.6	90.6	79.6	-12.2
	Piperacillin-tazobactam	13.8	14.2	+3.0	13.8	16.9	+22.7	13.6	15.3	+12.6	13.6	11.8	-12.9
	Broad-spectrum	23.4	26.3	+12.2	22.6	31.3	+38.4	23.2	28.7	+23.9	22.4	21.1	-5.8

Hospital	antibiotic (group)	Total			1st wave			2nd wave			Int. period		
		PC	C	%	PC	C	%	PC	C	%	PC	C	%
	Pneumonia AB[1]	42.0	43.0	+2.2	37.8	51.5	+36.2	41.5	43.6	+5.0	44.2	40.0	-9.6
	Carbapenems	6.77	8.57	+26.6	6.70	9.05	+35.0	6.54	9.79	+49.7	6.41	6.48	+1.0
	Fluoroquinolones	1.59	1.51	-5.1	2.59	3.60	+38.8	1.17	1.59	+35.5	1.73	1.22	-29.4
	Macrolides	8.53	6.19	-27.4	10.5	9.83	-6.4	8.92	5.62	-36.9	8.22	5.26	-35.9
	Azithromycin	1.23	2.74	+122.7	0.00	1.75	NA	1.18	4.25	+260.8	0.33	-2.09	-728.9
	Co-Amoxicillin	16.4	16.5	+0.2	15.0	20.5	+36.7	16.2	15.7	-3.1	17.1	16.7	-2.5
	3rd and 4th generation Cephalosporins	9.76	11.3	+16.0	7.61	13.4	+76.4	9.57	12.4	+29.5	10.4	9.25	-10.7

[1]All antibiotics recommended in the SSI guidelines (SSI 2022) for the treatment of pneumonia (doxycycline, amoxicillin-clavulanic acid, clarithromycin, ceftriaxone, levofloxacin, moxifloxacin, piperacillin-tazobactam).

Table S4: *Detailed results for antibiotic consumption analyses of the monthly IQVIA data. The table shows consumption in each pandemic phase compared to the same periods before the pandemic.* Data source: *IQVIA sales data (Sell-in) from pharmaceutical industries to hospital sector (01/2018-6/2021) PC, pre-COVID-19 period; C, COVID-19 period; Int. period; intermediate period; AB, antibiotics; J01, antibacterials for systemic (ATC category J01).*

Linguistic region	antibiotic (group)	Total			1st wave			2nd wave			Int. phase		
		PC	C	%	PC	C	%	PC	C	%	PC	C	%
**Switzerland**	J01	1.58	1.33	-15.8	1.58	2.11	+33.4	1.60	1.26	-21.1	1.51	1.17	-22.3
	Piperacillin-tazobactam	0.06	0.06	-4.1	0.06	0.08	+36.5	0.07	0.07	+0.4	0.06	0.05	-12.6
	Broad-spectrum	0.12	0.12	0.0	0.11	0.16	+40.7	0.12	0.14	+14.4	0.11	0.10	-13.6
	Pneumonia AB[1]	0.82	0.71	-13.6	0.82	1.23	+49.9	0.83	0.65	-21.5	0.76	0.61	-19.6
	Carbapenems	0.05	0.05	-0.1	0.04	0.07	+61.2	0.05	0.05	+4.9	0.05	0.04	-18.7
	3rd/4th generation Cephalosporins	0.15	0.15	+1.8	0.14	0.19	+36.3	0.15	0.15	+2.2	0.14	0.13	-6.3
	Fluoroquinolones	0.04	0.03	-23.7	0.04	0.06	+51.7	0.04	0.03	-33.0	0.03	0.02	-33.9
	Macrolides	0.09	0.07	-16.4	0.10	0.27	+175.1	0.09	0.05	-41.9	0.07	0.04	-47.3

Linguistic region	antibiotic (group)	Total			1st wave			2nd wave			Int. phase		
		PC	C	%	PC	C	%	PC	C	%	PC	C	%
	Azithromycin	0.02	0.02	+4.4	0.02	0.11	+429.8	0.02	0.01	-35.4	0.02	0.01	-61.2
	Co-Amoxicillin	0.48	0.40	-16.7	0.48	0.68	+40.3	0.49	0.37	-24.5	0.46	0.36	-21.7
**French**	J01	1.37	1.23	-9.9	1.35	2.31	+71.8	1.40	1.17	-16.2	1.31	0.99	-24.3
	Piperacillin-tazobactam	0.06	0.06	-2.0	0.07	0.09	+33.4	0.06	0.06	+5.3	0.05	0.04	-14.3
	Broad-spectrum	0.12	0.12	0.0	0.12	0.18	+47.7	0.12	0.14	+23.4	0.11	0.10	-6.5
	Pneumonia AB[1]	0.71	0.64	-10.1	0.70	1.29	+84.7	0.73	0.59	-20.0	0.66	0.52	-21.4
	Carbapenems	0.05	0.05	+2.6	0.05	0.08	+52.9	0.05	0.05	+2.4	0.05	0.05	-12.2
	3rd/4th generation Cephalosporins	0.15	0.16	+4.1	0.14	0.20	+40.0	0.16	0.16	+2.6	0.15	0.15	-0.2
	Fluoroquinolones	0.05	0.04	-28.2	0.05	0.08	+42.8	0.05	0.04	-31.1	0.05	0.02	-48.6
	Macrolides	0.09	0.11	+25.9	0.09	0.60	+537.2	0.09	0.05	-40.7	0.07	0.03	-58.6
	Azithromycin	0.03	0.05	+88.6	0.03	0.28	+1023.2	0.03	0.03	+4.6	0.03	0.01	-61.5
	Co-Amoxicillin	0.37	0.33	-11.1	0.35	0.59	+68.9	0.38	0.31	-18.2	0.35	0.28	-19.7
**German**	J01	1.63	1.35	-17.0	1.65	1.96	+18.4	1.65	1.29	-22.2	1.57	1.25	-20.1
	Piperacillin-tazobactam	0.06	0.06	-5.4	0.06	0.07	+17.0	0.06	0.07	+0.3	0.05	0.05	-10.9
	Broad-spectrum	0.11	0.11	0.0	0.11	0.13	+19.7	0.11	0.13	+12.2	0.11	0.09	-12.3
	Pneumonia AB[1]	0.84	0.73	-13.7	0.85	1.16	+36.2	0.85	0.67	-20.8	0.80	0.66	-17.3
	Carbapenems	0.04	0.04	+0.3	0.04	0.05	+48.8	0.04	0.04	+5.9	0.04	0.04	-13.6
	3rd/4th generation Cephalosporins	0.14	0.14	+2.4	0.13	0.18	+31.8	0.14	0.14	+2.5	0.13	0.13	-6.0
	Fluoroquinolones	0.03	0.02	-18.1	0.03	0.05	+50.7	0.03	0.02	-31.7	0.03	0.02	-18.9
	Macrolides	0.08	0.06	-30.4	0.10	0.15	+51.1	0.09	0.05	-41.7	0.07	0.04	-40.9
	Azithromycin	0.02	0.01	-33.3	0.02	0.05	+167.9	0.02	0.01	-52.6	0.02	0.01	-60.8
	Co-Amoxicillin	0.53	0.44	-17.1	0.53	0.70	+30.7	0.53	0.40	-24.6	0.50	0.40	-20.8
**Italian**	J01	1.97	1.49	-24.3	1.91	3.53	+84.7	1.98	1.43	-28.0	1.74	0.93	-46.7
	Piperacillin-tazobactam	0.10	0.10	+2.9	0.08	0.32	+315.3	0.11	0.09	-16.7	0.09	0.06	-25.1
	Broad-spectrum	0.22	0.22	0.0	0.18	0.59	+228.9	0.22	0.24	+4.7	0.20	0.10	-49.6
	Pneumonia AB[1]	1.03	0.74	-27.9	0.99	1.96	+97.2	1.05	0.67	-36.3	0.87	0.46	-47.1

Linguistic region	antibiotic (group)	Total			1st wave			2nd wave			Int. phase		
		PC	C	%	PC	C	%	PC	C	%	PC	C	%
	Carbapenems	0.11	0.10	-10.2	0.10	0.27	+165.2	0.11	0.12	+7.0	0.11	0.03	-70.3
	3rd/4th generation Cephalosporins	0.29	0.26	-9.9	0.28	0.46	+62.6	0.29	0.28	-1.0	0.24	0.17	-31.9
	Fluoroquinolones	0.09	0.05	-39.7	0.09	0.17	+93.1	0.09	0.05	-46.4	0.07	0.02	-71.4
	Macrolides	0.10	0.06	-46.2	0.09	0.19	+110.6	0.11	0.05	-51.3	0.07	0.02	-72.4
	Azithromycin	0.04	0.02	-38.8	0.04	0.09	+159.0	0.04	0.02	-62.8	0.03	0.01	-63.3
	Co-Amoxicillin	0.49	0.31	-36.0	0.49	0.93	+91.3	0.50	0.24	-51.8	0.42	0.20	-51.9

[1]All antibiotics recommended in the SSI guidelines (SSI 2022) for the treatment of pneumonia (doxycycline, amoxicillin-clavulanic acid, clarithromycin, ceftriaxone, levofloxacin, moxifloxacin, piperacillin-tazobactam).

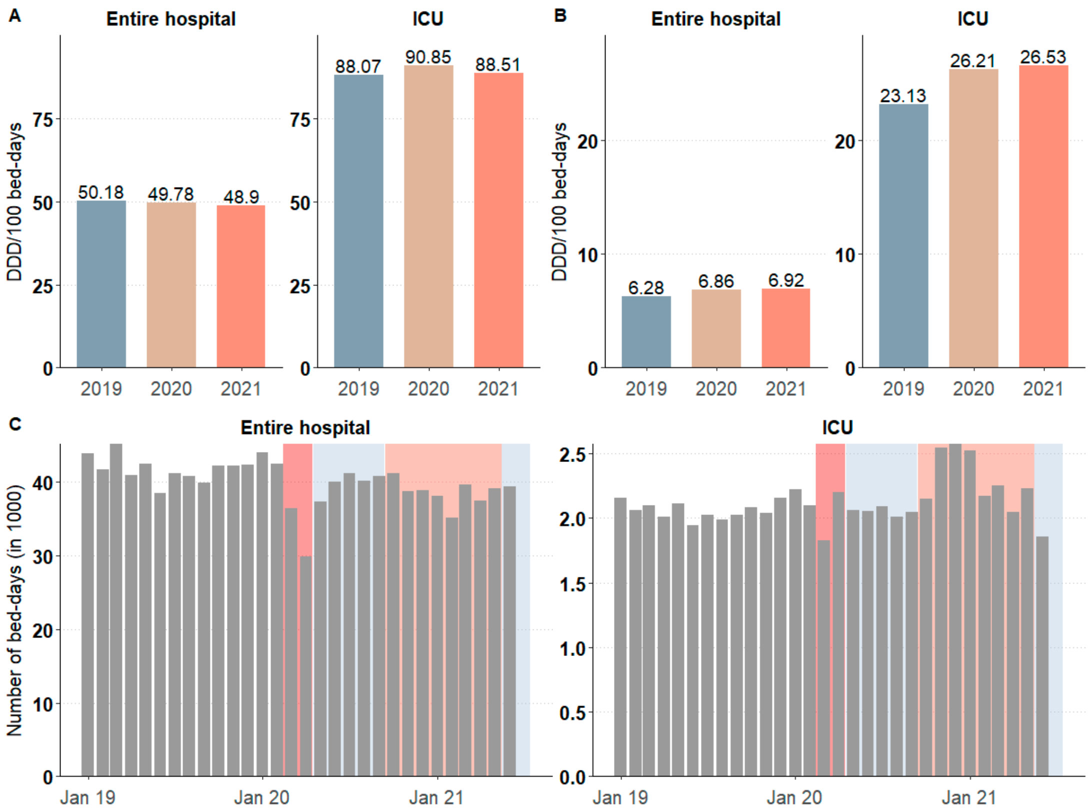

Figure S1 *Characteristics of the four hospitals submitting monthly antibiotic consumption and occupancy data for the entire hospital (left side) and ICU (right side). A: Antibiotic consumption (J01) for systemic use (ATC code J01) in DDD/100 bed-days, B) broad-spectrum antibiotics consumption in DDD/100 bed-days, C) hospital occupancy in thousands. ICU, intensive care unit.*

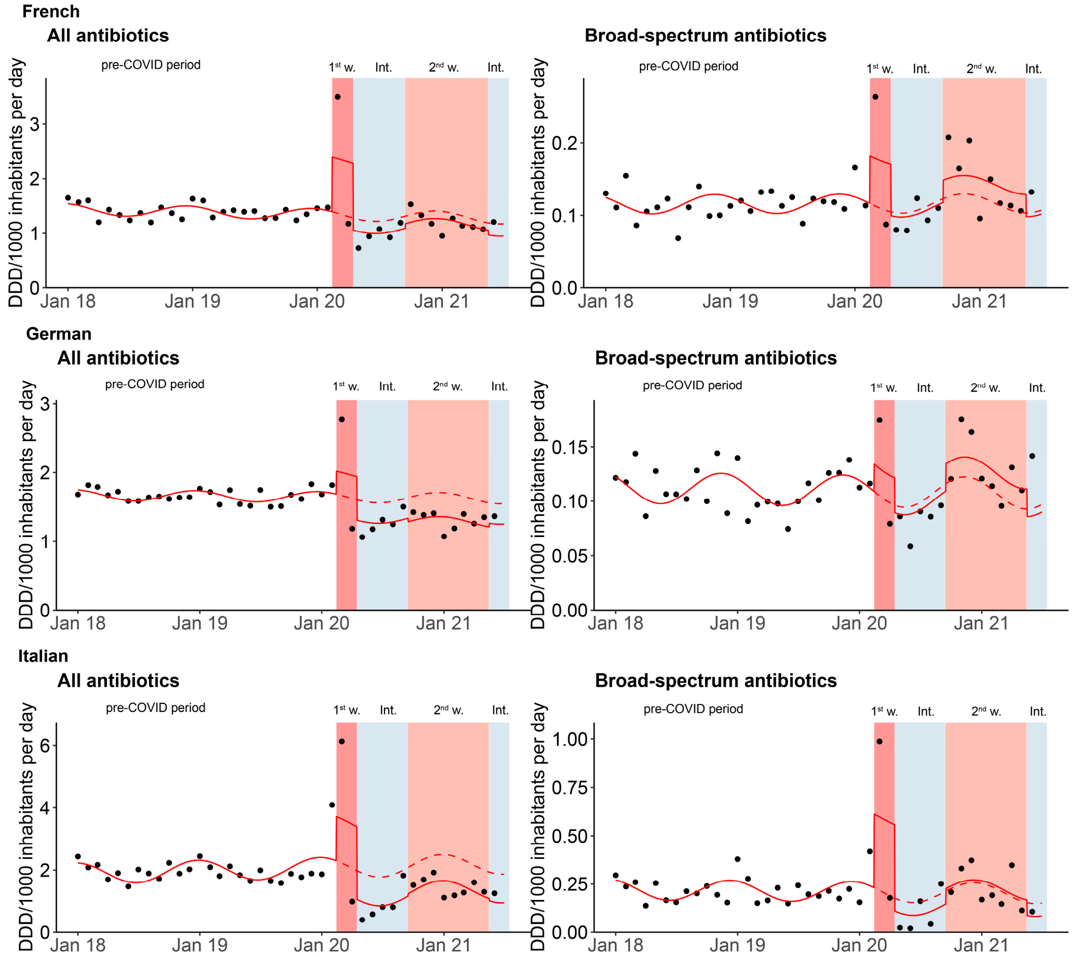

Figure S2 *Monthly antibiotic sales data measured in DDD per 1000 inhabitants per day of all antibiotics for systemic use (ATC code J01) between 01/2018 and 06/2021 in the French-, German-, and Italian-speaking Switzerland (IQVIA data). The plot shows the impact of each pandemic phase on antibiotic consumption based on segmented regression analysis. The solid line shows the estimates of the segmented regression model; the dotted line shows the estimated regression model assuming the pandemic had not occurred. The dots show the effective consumption per month.* Data source: IQVIA sales data (Sell-in) from pharmaceutical industries to hospital sector. *1st w, 1st wave; Int., intermediate phase; 2nd w, 2nd wave.*

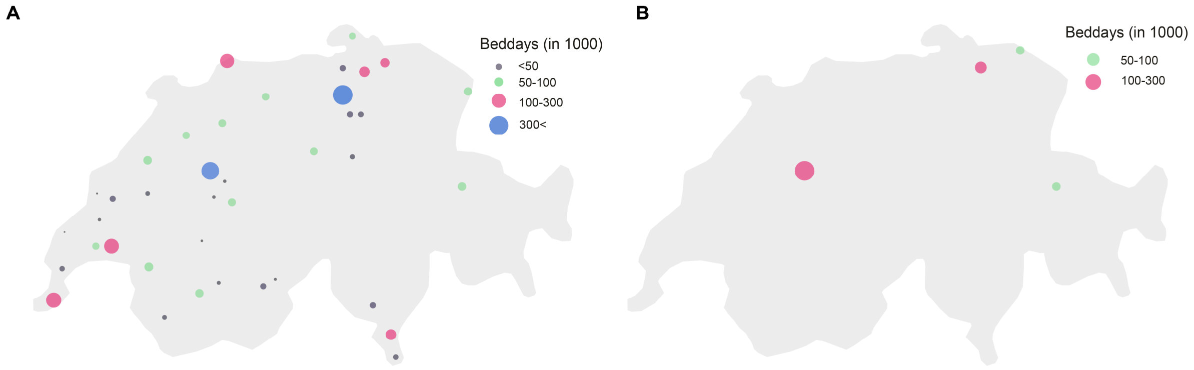

Figure S3 *A) Location of the hospitals included in the ANRESIS annual antibiotic consumption dataset. The size and color of dots reflect the range of aggregated number of bed-days per city (a city may have multiple hospitals). B) Location of the hospitals with monthly data that participated in ANRESIS antibiotic consumption monitoring. The size and color of the dots reflect the number of bed-days per hospital (ANRESIS monthly dataset).*
